# Exposure of *Drosophila melanogaster* to Mancozeb Induces Oxidative Damage and Modulates Nrf2 and HSP70/83

**DOI:** 10.1155/2018/5456928

**Published:** 2018-07-05

**Authors:** Miriane Acosta Saraiva, Eduardo da Rosa Ávila, Gustavo Felipe da Silva, Giulianna Echeverria Macedo, Nathane Rosa Rodrigues, Patrícia de Brum Vieira, Mariele Samuel Nascimento, Rochele Sogari Picoloto, Illana Kemmerich Martins, Nelson Rodrigues de Carvalho, Jeferson Luis Franco, Thais Posser

**Affiliations:** ^1^Oxidative Stress and Cell Signaling Research Group, Universidade Federal do Pampa, Campus São Gabriel, 97300-000 São Gabriel, RS, Brazil; ^2^Departamento de Medicina-Núcleo de Saúde, Universidade Federal de Rondônia, 76801-059 Porto Velho, RO, Brazil; ^3^Centro Interdisciplinar de Pesquisas em Biotecnologia (CIPBIOTEC), Universidade Federal do Pampa, Campus São Gabriel, 97300-000 São Gabriel, RS, Brazil; ^4^Departamento de Química (DQ), Centro de Ciências Naturais e Exatas (CCNE), Universidade Federal de Santa Maria, 97105-900 Santa Maria, RS, Brazil; ^5^Instituto Federal Farroupilha, Campus Santo Ângelo, 98806-700 Santo Ângelo, RS, Brazil

## Abstract

Mancozeb (MZ), a manganese- and zinc-containing ethylene-bis-dithiocarbamate, is a broad-spectrum fungicide. Harmful effects of this fungicide have been reported in nontarget organisms via a not fully understood mechanism. *Drosophila melanogaster* has provided remarkable contributions for toxicological studies. This work was aimed at evaluating the biochemical targets and implication of oxidative stress in MZ-mediated toxicity in drosophilas. Exposure of flies for fifteen days to MZ at 5 and 10 mg/mL through the diet impaired locomotor performance and induced fly mortality. In parallel, it caused lipid peroxidation and reactive oxygen species (ROS) formation and Mn overload. MZ inhibited superoxide dismutase and inducted catalase and glutathione S-transferase activities. Nitric oxide and reduced glutathione levels were significantly decreased by MZ. Heat shock proteins (HSP70 and HSP83) and Nrf2 mRNA levels were significantly augmented in MZ-exposed flies. Our study reinforced the use of *Drosophila melanogaster* as a reliable model for the study of biochemical targets of pesticides, and based on our data, MZ induced oxidative damage and Mn accumulation in a concentration-dependent manner. An adaptative cellular state was inducted by the lower concentration of pesticide, possibly contributing to the slighter damage observed.

## 1. Introduction

Mancozeb (manganese/zinc ethylene-bis-dithiocarbamate) is a broad-spectrum contact fungicide (MZ) that has been widely used in agriculture for controlling fungal infections in different crops such as soybean, tobacco, and ornamental plants [1]. MZ is categorized as mildly toxic for vertebrates; however, evidence from animal experimentation reported neurotoxicity, genotoxicity, and endocrine dysfunctions associated with exposure to this compound. MZ induced chromosomal aberrations and caspase activation in cultured human lymphocytes [2]; alteration in amino acid content in cerebellum and lower locomotor activity in pups exposed prenatally to MZ were reported [3]. Adult rats treated for prolonged periods with MZ presented hepatotoxicity and DNA damage with an augmented frequency of micronuclei [4, 5]. Deleterious effects of mancozeb were visualized in invertebrates as well, reducing the lifespan of butterflies and causing developmental defects [6]. Additionally, MZ decreased body and progeny and induced leg paralysis in fruit flies [7].

Mechanisms of action contributing to toxicological effects of MZ to vertebrates are not fully known, but the main line of evidence points to oxidative stress playing a significant role in MZ toxicology. Oxidative stress arises from an unbalance between reactive oxygen species (ROS) levels and cellular antioxidant defense systems able to neutralize them [8]. This condition may lead to oxidative damage to biomolecules such as proteins, lipids, and nucleic acids [9] and is implicated in the etiology of several diseases, such as tumors and neurodegenerative disorders [10–12]. A possible cause of the increment of ROS levels by MZ is the inhibition of the mitochondrial complexes activity, resulting in an inefficient transfer of electrons, leading to the formation of oxygen radicals [13, 14]. Also, induction of NADPH oxidase and xanthine oxidase by MZ leading to augmented H_2_O_2_ has been reported [15], and modulation of antioxidant enzyme activity, as published recently by our research group, has been reported in carp brain [16].

One potential mechanism by which MZ induces oxidative stress may be attributed to the chelating properties of these class of compounds toward transition elements in the structure of proteins leading to the formation of complexes and resulting in an inhibitory action, as reported for SOD [17].

Flies are considered a very sophisticated and complex model organism in scientific research. The adult flies have structures that share equivalent functions to the mammal's heart, lung, kidney, gut, and reproductive tract. Furthermore, flies display complex behaviors as circadian rhythms, learning, memory, and feeding among others. Likewise, the responses of flies to different drugs are very comparable with that observed in mammals providing an attractive alternative model in pharmacology and toxicology. Therefore, although evolutionarily distant, conserved aspects of biology and physiology position the fruit flies as a valuable tool in the description of mechanisms implied in chemicals toxicology [18–20].

This study is aimed at investigating the effects from consumption of MZ through the diet on survivorship; locomotor performance; and biochemical parameters as oxidative stress; modulation of activity; and mRNA levels of antioxidant enzymes, HSPs, and Nrf2 in fruit flies. Our results extend the knowledge on the biochemical action of MZ and reinforce the use of drosophilas in toxicological studies.

## 2. Materials and Methods

### 2.1. Reagents

Dimethylsulfoxide, quercetin, 5,5′-dithio-bis (2-nitrobenzoic acid), acetylcholine iodide, 1-chloro-2,4-dinitrobenzene, 2′,7′-dichlorofluorescein diacetate (DCHF-DA), Folin-Ciocalteu, HEPES minimum 99.5% titration, 2,4,6–tris(2-pyridyl)-5-triazine (TPTZ), resazurin sodium salt, albumin from bovine serum (BSA), sucrose, reduced glutathione, tetramethylethylenediamine, and quercetin were purchased from Sigma-Aldrich (São Paulo, SP, Brazil). DNAse I Amplification Grade, SYBR Select Master Mix, and TRIzol were obtained from Life Technologies; iScript cDNA Synthesis kit was obtained from Biorad (CA, USA). Griess reagent system and Caspase-Glo 3/7 and caspase 9 were purchased from Promega (WI, USA). Mancozeb (80% purity, Enzeb 800 WP) was purchased from Sabero Organics America SA (Belo Horizonte, MG, Brazil). All other reagents were commercial products of the highest purity grade available.

### 2.2. Drosophila Culture and Procedures

Wild-type *Drosophila melanogaster* (Harwich strain) was obtained from the National Species Stock Center, Bowling Green, OH, USA. The flies were cultured under controlled temperature of 25 ± 1°C, 12 h dark-light photoperiod, and 50–60% relative humidity. The standard cornmeal diet was composed of 39% cereal flour, 32% corn flour, 14% glucose, 2% powdered milk, 1% salt, 10% distilled water, and 1% antifungal agent (Nipagin®) and supplemented with 1% dried yeast as previously described by Macedo et al. [21].

### 2.3. Exposure of Flies to Mancozeb

Adult male fruit flies 2 days posteclosion were exposed to different concentrations of MZ (0, 5, and 10 mg of commercial MZ powder per mL of standard diet) for 15 days in polypropylene vials. Each vial contained 60 individuals. The concentrations of mancozeb used in this study were based on the field rate dilution recommended by the manufacturer (Sabero Organics America SA, Brazil) for fruit crops. This concentration is also by the recommendation of the United States Environmental Protection Agency [1]. Other studies have chosen a similar concentration to test the effect of mancozeb on worms [22].

### 2.4. Mortality and Locomotor Performance Assay

For mortality curve, the number of dead flies was registered daily for up to 15 days. The mobility assay was assessed by the Negative Geotaxis Test [23] at 7, 10, and 15 days of exposure. In brief, 6 groups of 10 flies per treatment were immobilized on ice for 1 minute and placed on glass tubes. After recovery, flies were gently tapped to the bottom of the glass column, and the number of flies that reach 5 cm of the column (top) was counted. Each group of flies was tested three times at 1-minute intervals.

### 2.5. Resazurin Reduction Assay

The conversion of resazurin to a fluorescent compound was used as a general index of metabolic activity [24]. For this test, twenty flies were homogenized in 1 mL of 20 mM Tris buffer, pH 7.0, and centrifuged at 1000 ×g for 10 minutes at 4°C. The supernatant was incubated in an ELISA plate containing buffer and 0.2 mg/mL resazurin for 4 h. The fluorescence was read in a multimode EnsPire® reader (Perkin Elmer 2300, Waltham, MA) at an excitation wavelength of 573 nm and emission of 584 nm. Results were expressed as percent fluorescence arbitrary units (mean ± SEM).

### 2.6. Caspase 3/7 and 9 Activity

To evaluate caspase activity, groups of twenty flies were homogenized in 20 mM HEPES buffer, pH 7.0, centrifuged at 2000 ×g for 1 minute at 4°C. The supernatant was used to quantify the caspase 3/7 and 9 using commercial kits (Apo-one homogeneous caspase 3/7 assay® and Caspase-Glo®; Promega, Madison, WI). The results were expressed as fluorescence/luminescence arbitrary units (mean ± SEM).

### 2.7. Determination of Arbitrary Steady-State ROS Levels and Nitric Oxide

The fluorescent dye 2,7-dichlorofluorescein diacetate (DCF-DA) was used to determine the arbitrary steady-state ROS level. After 15 days of treatment, twenty flies were homogenized in 20 mM Tris buffer, pH 7.0. The homogenate was centrifuged at 1600 ×g for 10 minutes at 4°C, and the supernatant was removed for quantification of 2,7-dichlorofluorescein fluorescence as previously described by Pérez-Severiano et al. [25] and was monitored after one hour at 488_nm_/530_nm_ excitation/emission in a multimode reader. The results were expressed as arbitrary fluorescence units (mean ± SEM). Nitric oxide was evaluated by the measurement of nitrite (NO_2_^−^) levels using the Griess reagent system protocol (Promega® Madison, WI). Briefly, supernatants were distributed to 96-well plates in triplicates in the presence of a Griess reagent (1% sulfanilamide, 0.1% N-(1-naphthyl) ethylenediamine dihydrochloride, and 5% H_3_PO_4_). After incubation at room temperature, the optical density (OD) was read at 540 nm using a multimode reader. The NO_2_^−^ concentration was calculated from a standard curve generated with the nitrite standard ranging from 0 to 100 *μ*M.

### 2.8. Malondialdehyde (MDA) Quantification

The determination of MDA was played by HPLC using a Shimadzu Prominence UFLC with LC-6AD contained absorbance detector UV SPD-20AV. The software LC Solution was applied for analyzing retention time, chromatograms, and its evaluation. The column utilized was reverse phase Kromasil® C18 (250 mm × 4.6 mm, d.i.; 5 *μ*m). To prepare the samples, twenty male flies were homogenized in a bead-based PowerLyser® homogenate with 150 *μ*L of 0.5 M HClO_4_ and 150 *μ*L of distilled water, and the homogenate was centrifuged (10,000 ×g for 10 minutes at 4°C). The supernatant was injected into the HPLC as described by Karatas et al. 2002 [26], with minor modifications. The flux was modified to 0.8 mL/min^−1^ with average time retention of 3.528 minutes. The heads of flies were previously removed to avoid the interference of eye pigments with MDA absorbance. Results were expressed as *μ*g/mL (mean ± SEM).

### 2.9. Determination of Thiol Levels

The measurement of thiol levels was performed by the reaction of −SH group-containing species present in the cells with O-phthaldehyde as described by Hissin and Hilf [27]. For this technique, 20 flies were homogenized in 100 mM sodium phosphate buffer, pH 8.0, containing 5 mM EDTA and 5% phosphoric acid and then centrifuged at 100,000 ×g in an ultracentrifuge (HITACHI CP-100WX), for 30 min at 4°C. The supernatant was incubated in the presence of 1 mg/mL O-phthalaldehyde. The fluorescence was measured at 350_nm_/420_nm_ excitation/in a multimode plate reader.

### 2.10. Enzyme Activity Assay

Twenty flies were homogenized in 20 mM HEPES, pH 7.0, and centrifuged for 1000 ×g for 5 minutes at 4°C. A supernatant aliquot was separated for acetylcholinesterase (AchE) activity assay according to Ellman [28]. The remaining supernatant was centrifuged at 20,000*g* for 30 min at 4°C to measure the activity of catalase (CAT), glutathione S-transferase (GST), and superoxide dismutase (SOD). The CAT activity was determined following the clearance of H_2_O_2_ at 240_nm_ in a reaction media containing 50 mM phosphate buffer, pH 7.0, 0.5 mM EDTA, 10 mM H_2_O_2_, and 0.012%Triton X100 as described by Aebi [29]. The GST activity was measured based on the GST-driven reaction of GSH with the substrate 1-chloro-2,4-dinitrobenzene (CDNB), leading to the formation of a complex of CDNB and GSH at 340 nm according to Habig and Jakoby [30]. The mixture was composed of 100 mM phosphate buffer (pH 7.0), 1 mM EDTA, 1 mM GSH, and 2.5 mM CDNB. The assay for SOD activity quantification consists of the inhibition of superoxide-driven oxidation of quercetin by SOD at 406_nm_ according to Kostyuk and Potapovich [31]. The reaction system consisted of 25 mM phosphate buffer, pH 10, 0.25 mM EDTA, 0.8 mM TEMED, and 0.05 *μ*M quercetin. The enzymatic activities were calculated in milliunits per milligram of the total protein content, which was quantified by Bradford assay [32] using bovine serum albumin (BSA) as the standard. Results are expressed in % relative to the control group.

### 2.11. Metal Content

For verification of metal levels in *D. melanogaster*, male flies until 48 hours post-eclosion were exposed for fifteen days at two different MZ concentrations (5 mg/mL and 10 mg/mL). After the exposition, two hundred flies per group including the control were washed three times with distilled water abundantly and dried out for 90 minutes at 37°C. After this process, an inductively coupled plasma optical emission spectrometer (Optima 4300 DV, PerkinElmer, USA) with axial view configuration was used for the determination of calcium (Ca), copper (Cu), iron (Fe), potassium (K), magnesium (Mg), manganese (Mn), sodium (Na), phosphorus (P), sulfur (S), and zinc (Zn). Argon (99.998%, White Martins-Praxair, Brazil) was used for plasma generation, nebulization, and auxiliary gas. The operational conditions were set according to work described previously by Muller et al. [33]. The metal content was expressed relative to the weight of samples used for analysis (*μ*g metal/g of dried weight sample). Before determination, flies were digested in closed vessels by the work described by Bizzi et al. [34]. Samples (approximately 60 mg) were transferred directly into the closed quartz vessels, and 6 mL of nitric acid 2 mol·L^−1^ was added. After closing the vessels, and capping of the rotor, each vessel was pressurized with oxygen at 7.5 bar. Then, the rotor was placed inside a microwave oven (Multiwave 3000 Microwave Sample Preparation System, Anton Paar, Graz, Austria). The system was equipped with eight high-pressure quartz vessels (80 mL, maximum pressure and temperature of 80 bar and 280°C, resp.). The microwave-heating programs used were (a) 1000 W, with a ramp of 5 min; (b) 1000 W for 10 min; and (c) 0 W for 20 min (cooling step). The pressure and temperature were monitored in each vessel during the run. The resultant solutions were diluted with water up to 25 mL in volumetric flasks. After each run, vessels were cleaned using 6 mL of concentrated HNO_3_, and the microwave programs were 1400 W for 10 min and 0 W for 20 min (cooling), and after, vessels were also rinsed with water. Three readings were averaged to give one value per biological replicate and expressed as a mean ± standard deviation of the mean (SD). Metal levels were expressed relative to the weight of flies used for analysis (*μ*g metal/g of dried weight tissue).

### 2.12. Relative Steady-State Level of mRNA

The profile of the relative steady-state level of specific mRNA in response to the treatments was performed by the polymerase chain reaction from the reverse transcriptase (RTq-PCR). Around 1 *μ*g of the total RNA from 20 adult male flies was extracted using the TRIZOL Reagent (Invitrogen®, CA, EUA) according to the manufacturer's protocol. The relative steady-state level of mRNA of GPDH, HSP70, HSP83, CAT, SOD, and Nrf2 (Table 1) was analyzed. The total RNA was treated with DNase I (DNAse I Amplification Grade—Invitrogen CA, EUA), and cDNA was produced with iScript cDNA Synthesis Kit. Quantitative real-time polymerase chain reaction was performed according to what was previously described by Macedo et al. [21].

### 2.13. Statistical Analysis

Lifespan measurement was determined by comparing the survival curves with a log-rank (Mantel-Cox) test. Another statistical analysis was performed using one-way ANOVA and Tukey's posthoc test. Differences were considered significant between groups at *P* < 0.05.

## 3. Results

### 3.1. Mancozeb Exposure Reduces the Lifespan of *D. melanogaster* and Impairs Locomotor Performance

**Figure 1:**
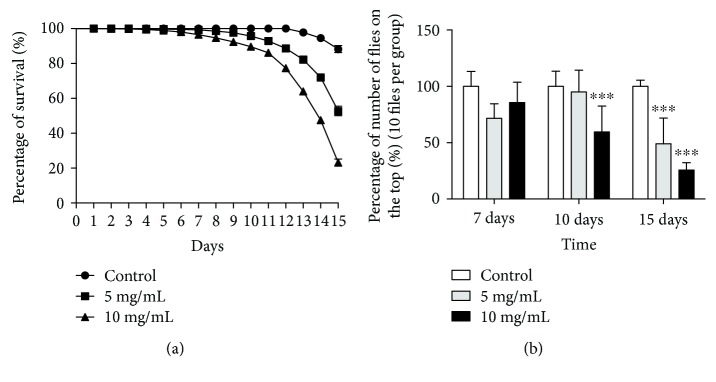
Effects of MZ exposure on survivorship, negative geotaxis. (a) The number of survival flies exposed to MZ was daily registered and expressed as percentage of survival flies with respect to the control group (ten flies were evaluated; an experiment was performed in triplicate). The statistic was performed by comparing the survival curves with a log-rank (Mantel-Cox) test. (b) After 15 days of exposure to MZ, negative geotaxis was evaluated. Results are presented as means ± SEM, *P* < 0.001 from 3 different preparations. ^∗∗∗^*P* < 0.001.

To evaluate the effects of MZ in fruit flies, we have determined the mortality curve and locomotor performance (Figures 1(a) and 1(b), resp.). After 15 days of exposure, MZ caused 80% of mortality at highest concentration (10 mg/mL) and 53% of mortality to intermediate concentration (5 mg/mL). At the 7th day of exposure, the impairment in locomotor deficits was visualized at 5 and 10 mg/mL (*P* < 0.0001). This effect was intensified over time, and after 15 days of exposure to the highest concentrations, surviving flies were totally unable to climb and remained at the base of the tube (Figure 1(b)). All the biochemical assays were performed after 15 days of MZ. This period of treatment was chosen because the most significant effects on fly mortality were observed after 15 days; moreover, the manufacturer (Sabero Organics America SA, Brazil) recommends the field reapplication of intervals of 15 days after the first application, totalizing 4 applications per cycle.

### 3.2. Cell Viability and Caspase 3/7 and 9 Activity

**Figure 2:**
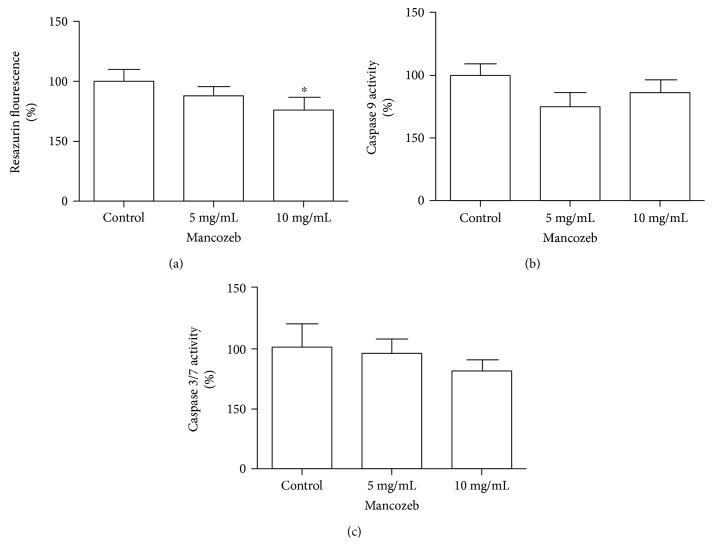
Cellular viability and caspase 3/7 and 9 activity in response to 15 days of MZ treatment. (a) Resazurin fluorescence, (b) caspase 9 and (c) caspase 3/7 were evaluated in flies exposed to MZ. All experiments are expressed in percentage in relation to the control and represent an average of three experiments performed in triplicate. Results are presented as means from 3 different preparations. ^∗^*P* < 0.05.

A reduction of 12% (*P* < 0.0001, *F* = 13.99) in the cellular viability by Resazurin reduction assay was observed, at a concentration of 10 mg/mL after 15 days of treatment (Figure 2(a)). The treatment with MZ did not alter significantly the activity of caspase 3/7 and 9 (*P* = 0.1730, *F* = 2.146 and *P* = 0.051, *F* = 5.919, resp.) (Figures 2(b) and 2(c)).

### 3.3. Lipid Peroxidation, ROS, and NO Levels in Response to MZ Exposure

**Figure 3:**
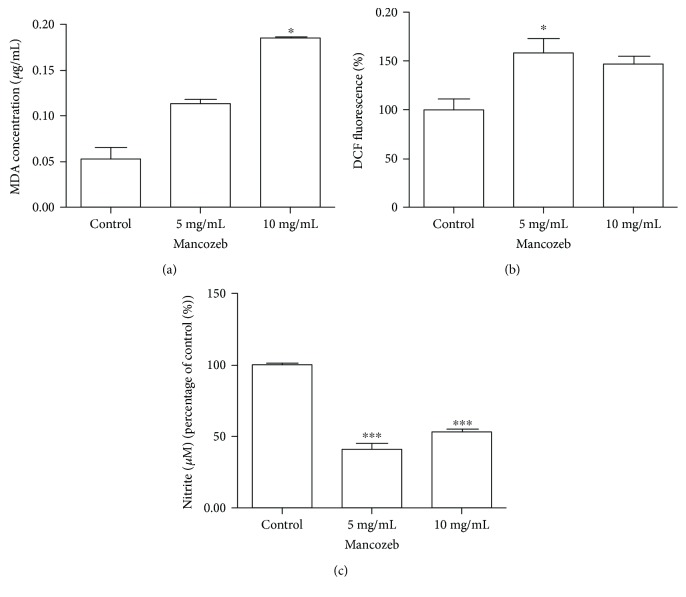
Effects of MZ exposure on MDA levels, arbitrary steady-state ROS levels, and nitric oxide levels. (a) MDA levels were evaluated after 15 days of exposure by HPLC on a fly homogenate. (b) ROS levels were evaluated by DCF-DA fluorescence. (c) Nitric oxide production was evaluated by a colorimetric assay based on mitrite presence. All experiments were repeated three times and performed in triplicate. ^∗^*P* < 0.05 and ^∗∗∗^*P* < 0.0001. Results are presented as means ± SEM, from 3 different preparations.

Lipid peroxidation was quantified in the whole-body homogenate of flies by MDA quantification (Figure 3(a)). The highest concentration of MZ induced a fourfold increase in MDA content (*P* < 0.0001, *F* = 236.6). In parallel, the ROS levels in fly homogenate were investigated (Figure 3). A reactive species was produced by MZ treatment from 5 mg/mL (58%), whereas the NO amount quantified by nitrite levels (NO^−^_2_) was 59% and 47% lower than the control group at concentrations of 5 and 10 mg/mL, respectively (*P* < 0.0001, *F* = 139.1) (Figure 3(c)).

### 3.4. Antioxidant Enzyme Activity and GSH Levels in Flies Exposed to MZ Treatment

**Figure 4:**
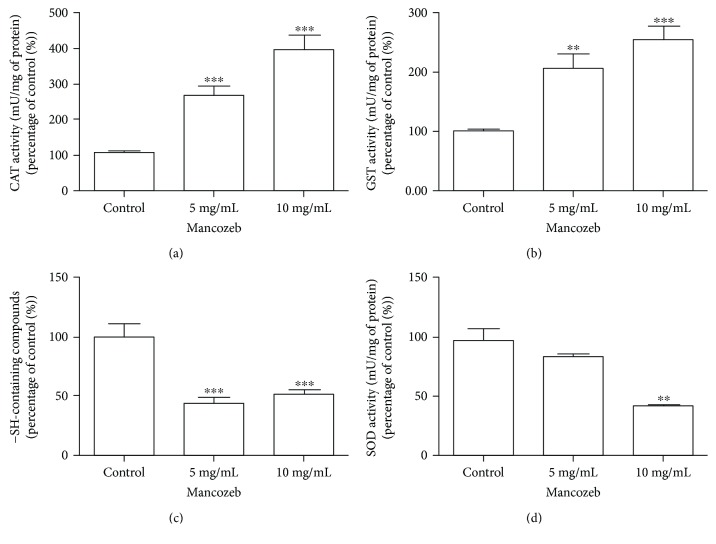
Effects of MZ exposure on the CAT, GST, and SOD activity and −SH group-containing species after 15 days of exposure in *D. melanogaster.* (a) CAT, (b) GST, (c) −SH group-containing species levels, and (d) SOD activity were measured in the whole homogenate of flies. The activity of antioxidant enzymes is expressed in percentage in relation to control group. All experiments were reiterated three times and performed in triplicate. ^∗∗^*P* < 0.05 and ^∗∗∗^*P* < 0.001 in relation to control. Results are presented as means ± SEM, from 3 different preparations.

The enzymes catalase (CAT), glutathione S-transferase (GST), and superoxide dismutase (SOD) and levels of reduced glutathione (GSH) were evaluated. The treatment with MZ stimulates CAT activity in 170% at 5 mg/mL and 300% at 10 mg/mL (*P* < 0.0001, *F* = 87.59) (Figure 4(a)). GST activity was increased by 100% and 154% at 5 mg/mL and 10 mg/mL, respectively (*P* < 0.0001, *F* = 16.76) (Figure 4). In contrast, the activity of SOD was inhibited by 66% only at the highest concentration (10 mg/mL) of MZ (Figure 4(d)) without alteration in the lower concentration (*P* < 0.005, *F* = 41.35). Thiol levels were 33% lower than those in the control group in both concentrations analyzed (*P* < 0.0001, *F* = 64.43) (Figure 4(c)).

### 3.5. Measurement of Metal Levels in Response to MZ in *D. melanogaster*

Levels of Mn and other essential metals were evaluated by inductively coupled plasma optical emission spectrometer (ICPOES). Our results showed a concentration-dependent increase in Mn levels (0.11 and 0.19 mg/g at 5 mg/mL and 10 mg/mL, resp.) compared to control group (0.02 mg/g). There were no significant changes in the concentration of calcium (Ca), iron (Fe), potassium (K), magnesium (Mg), sodium (Na), potassium (P), sulfur (S), zinc (Zn), and copper (Cu) (Table 2).

### 3.6. Relative Steady-State Level of mRNA of Antioxidant Enzymes, Nrf2 Transcription Factor, and HSP70 and HSP83

**Figure 5:**
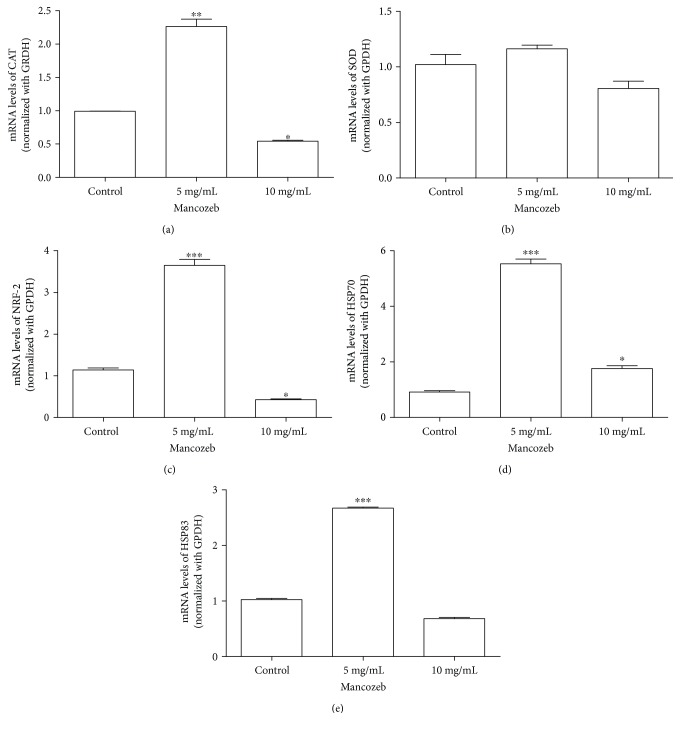
Relative steady-state level of specific mRNA in response to MZ exposure. The relative steady-state levels of the mRNA of (a) Cat, (b) SOD (c) Nrf2, (d) HSP70, and (e) HSP83 were evaluated after 15 days of exposure to MZ by RT-qPCR. All experiments were repeated three times and performed in triplicate. The data were normalized with GPDH constitutive gene. ^∗^*P* < 0.05; ^∗∗^*P* < 0.01; and ^∗∗∗^*P* < 0.001. Results are presented as means ± SEM, from 3 different preparations.

Considering the significant increase in the antioxidant enzyme activity, there was speculation about a possible alteration in the relative steady-state levels of mRNA of those genes related to the antioxidant enzymes (CAT and SOD). Additionally, the relative steady-state levels of mRNA of Nrf2 and heat shock proteins HSP83 and HSP70, which are molecular pathways closely related to cellular redox balance and cell adaptation to stressful conditions, were evaluated. Our results pointed to a concentration-dependent alteration in the relative steady-state level of the mRNA of analyzed genes. The relative steady-state levels of the mRNA of HSP70 (*P* < 0.005, F 430.7), HSP83 (*P* < 0.0001, *F* = 2359), CAT (*P* < 0.001, *F* = 19.21), and Nrf2 (*P* < 0.0005, *F* = 377.7) transcriptional factor were increased fivefold, twofold, twofold, and threefold, respectively, in relation to the control group at 5 mg/mL without significant alteration in the SOD mRNA level. At 10 mg/mL, a significant reduction in the relative steady-state level of mRNA for all targets' with the exception of SOD SOD (*P* = 0.07, *F* = 6.893) was observed at 10 mg/mL (Figures 5(a) and 5(e)).

## 4. Discussion

The present study was aimed at analyzing biochemical alterations resulting from the exposure of fruit flies *Drosophila melanogaster* up to 15 days to a diet mixed with MZ. Previous studies have reported lifespan shortening and progeny reduction of flies by MZ [7]; however, at least to our knowledge, this is the first study describing molecular targets and mechanisms of toxicity of this compound on fruit flies.

MZ induced an accumulation of Mn in a concentration-dependent manner in the flies. In recent study, MZ caused Mn accumulation in carp brain [16]. Mn is one of the main byproducts of MZ degradation, and accumulation of Mn in specific brain regions is related to neurotoxicity and behavioral symptoms similar to Parkinson's disease in humans and rodents [24, 35, 36]. Similarly, exposure to Mn induced locomotor deficits in flies [36–38]. It was demonstrated previously that the link of manganese (Mn) to the organic molecule of MZ accounts for toxicity of this compound [15]. Oxidative stress is proposed to be the main mechanism implied in Mn-induced injury. Different sources of ROS by Mn includes the oxidation of Mn^2+^ to Mn^3+^, which catalyzes DA oxidation with the formation of toxic and reactive intermediaries [39]. Also, Mn can increase the proportion of Fe (II), which can then prompt oxidative stress via the Fenton reaction [40]. Thus, the accumulation of Mn caused by MZ exposure, as measured by ICP-OS, could contribute to oxidative damage and behavioral impairment in flies by some of the mentioned mechanisms.

MZ stimulated GST activity; this effect was also described in rat hepatic tissue and carp brain [16, 41]. The family of protein GST plays multiple roles including cell protection against oxidative stress and potentially toxic compounds via conjugation of glutathione (GSH) to various molecules and products of oxidative metabolism [42], decreasing the toxicity and contributing to their elimination from the cell. An augmented GST activity was observed in both concentrations of MZ and occurred in parallel with decreased and reduced glutathione, indicating the activation of the detoxification mechanism mediated by GST in flies.

Since primitive life forms, SOD and catalase are primary ROS removal enzymes. SOD catalyzes the dismutation of superoxide (O_2_^°−^) to hydrogen peroxide (H_2_O_2_), a toxic product that must be quickly removed from the cell, and catalase detoxifies H_2_O_2_, reducing it to water and oxygen [10]. In this work, MZ inhibited the activity of SOD significantly, and similar results were observed in carp brain and human erythrocytes exposed to MZ [16, 43]. The chelating ability of dithiocarbamates toward metals present in the active center of enzymes contributes to enzymes inhibition, as was reported for SOD [17]. As no alteration in the SOD relative steady-state level of mRNA was observed in this study, a posttranslational inhibition of SOD due to an interaction with MZ molecule might be suggested. An unbalance of the antioxidant system resulting from SOD inhibition might cause augmented levels of O_2_^°−^ which, in turn, may react with °NO, generating peroxynitrite, a potent oxidizing compound that has an effect upon mitochondrial function and triggers cell death via oxidation and nitration reactions [44]. This hypothesis could explain why nitric oxide levels were decreased and may be an outcome of SOD inhibition (evidenced in this study) potentiating the pro-oxidant effect of MZ, contributing to oxidative damage to biomolecules, as indicated by MDA, a product of lipid peroxidation.

Nrf2 (nuclear factor erythroid 2- (NFE2-) related factor 2) is highly conserved across vertebrates, and similarly to mammals, in drosophilas, this factor regulates constitutive expression and coordinated induction of numerous gene encoding antioxidants and phase-2 detoxifying enzymes and related proteins, such as superoxide dismutases (SODs), catalase, UDP-glucuronosyltransferase, NAD(P)H:quinone oxidoreductase-1 (NQO1), heme oxygenase-1, glutamate cysteine ligase, glutathione S-transferase, glutathione peroxidase, and thioredoxin [43]. Nrf2 is implied in the promotion of cell resistance to chemicals, such as herbicide paraquat and arsenic [45, 46].

Heat shock proteins are reported to protect the cells against the damage promoted by oxidative stress [47–50]. These proteins assist in the correct folding of nascent and misfolded proteins. HSP genes are known as “stress genes,” serving as indicators of the cellular toxicity of different environmental stressors [47–50]. In this study, larger relative steady-state levels of the specific mRNA of Nrf2, catalase, HSP70, and 83 were observed at the concentration of 5 mg/mL, suggesting an adaptative response of flies to the presence of the lower concentration of MZ in the diet for 15 days [42, 43]. In contrast, at a higher concentration, a decrease in the relative steady-state level of the mRNA of those genes occurred, and this inhibition was not extended to the activity of catalase, which remained augmented. This contradictory result could be explained by taking into account the stability of proteins in comparison with mRNA. It was demonstrated previously that under oxidative insult, protein levels of SOD and other antioxidant proteins increased during the first 30 min and remained constant or slightly decreased over the time, while the mRNA decreased after 30 min of the oxidant insult [51]. These data point to superior protein stability or even an increased rate of translation per available mRNA. In this aspect, the higher activity of catalase observed here might represent a temporal event that will cause a diminished expression and activity of antioxidant enzymes and HSPs over the time.

## 5. Conclusion

Our study shows that MZ intake induces a time- and concentration-dependent locomotor impairment and fly death, which occurred in parallel with oxidative stress revealed by decreased thiol levels and altered activity enzymes culminating in oxidative damage to membranes and consequent cell death. The degree of insult was intensified at the highest concentration of MZ. This fact may be attributed to an adaptive response represented by the augmented expression of HSPs and Nrf2 gene, providing cytoprotection. These data draw attention to the hazardous effects of MZ on fruit flies and describe molecular targets of MZ on this model pointing to antioxidant and stress-related proteins as key factors on the adaptative response during MZ exposure.

## Figures and Tables

**Table 1:** Genes analyzed by quantitative real-time RT-qPCR in *D. melanogaster* exposed to MZ.

Genes	Primer sequences
GPDH	Left 5′-ATGGAGATGATTCGCTTCGT
	Right 5′-GCTCCTCAATGGTTTTTCCA

Catalase	Left 5′-ACCAGGGCATCAAGAATCTG
	Right 5′-AACTTCTTGGCCTGCTCGTA

HSP70	Left 5′-GCTGACGTTCAGGATTCCAT
	Right 5′-CGGAGTCTCCATTCAGGTGT

HSP83	Left 5′-CAAATCCCTGACCAACGACT
	Right 5′-CGCACGTACAGCTTGATGTT

Nrf2	Left 5′-CGTGTTGTTACCCTCGGACT
	Right 5′-AGCGCATCTCGAACAAGTTT

Superoxide dismutase	Left 5′-GGAGTCGGTGATGTTGACCT
	Right 5′-GTTCGGTGACAACACCAATG

**Table 2:** Metal contents in *D. melanogaster* exposed for 15 days at two different mancozeb concentrations.

Metals	Control	5 mg/mL	10 mg/mL
Ca	0.44 ± 0.04	0.41 ± 0.02	0.41 ± 0.08
Fe	0.34 ± 0.05	0.26 ± 0.03	0.22 ± 0.04
K	5.03 ± 0.56	4.56 ± 0.59	4.75 ± 0.53
Mg	0.59 ± 0.07	0.48 ± 0.07	0.54 ± 0.08
Mn	0.02 ± 0.01	0.11 ± 0.02	0.19 ± 0.02^∗^
Na	1.60 ± 0.14	1.64 ± 0.15	1.41 ± 0.24
P	5.76 ± 0.85	5.24 ± 0.71	6.05 ± 0.83
S	2.82 ± 0.36	2.62 ± 0.34	2.94 ± 0.38
Zn	0.09 ± 0.01	0.06 ± 0.01	0.09 ± 0.02
Cu	<QL	<QL	<QL

Effect of exposure to MZ during 15 days on metal levels of fruit flies. The value is shown in mg·g^−1^. Results are expressed as a mean ± standard deviation (*n* = 3). ∗ indicates significant difference in comparison to the control group (*P* < 0.05). The quantification limit (QL) for Cu is 0.0025 mg·g^−1^.

## Data Availability

The data are under the responsibility of the research group and can be accessed if requested.
